# Metabolic differences in ripening of *Solanum lycopersicum* ‘Ailsa Craig’ and three monogenic mutants

**DOI:** 10.1038/sdata.2014.29

**Published:** 2014-09-16

**Authors:** Stephan Beisken, Mark Earll, Charles Baxter, David Portwood, Zsuzsanna Ament, Aniko Kende, Charlie Hodgman, Graham Seymour, Rebecca Smith, Paul Fraser, Mark Seymour, Reza M. Salek, Christoph Steinbeck

**Affiliations:** 1 European Molecular Biology Laboratory—European Bioinformatics Institute (EMBL-EBI), Wellcome Trust Genome Campus, Hinxton, Cambridge CB10 2HA, UK; 2 Syngenta Jealott’s Hill International Research Centre, Bracknell, Berkshire RG42 6EY, UK; 3 Centre for Plant Integrative Biology, University of Nottingham, Loughborough, Leicestershire LE12 5RD, UK; 4 School of Biological Sciences, Royal Holloway, University of London, Egham Hill, Egham, Surrey TW20 0EX, UK

## Abstract

Application of mass spectrometry enables the detection of metabolic differences between groups of related organisms. Differences in the metabolic fingerprints of wild-type *Solanum lycopersicum* and three monogenic mutants, *ripening inhibitor* (*rin*), *non-ripening* (*nor*) and *Colourless non-ripening* (*Cnr*), of tomato are captured with regard to ripening behaviour. A high-resolution tandem mass spectrometry system coupled to liquid chromatography produced a time series of the ripening behaviour at discrete intervals with a focus on changes post-anthesis. Internal standards and quality controls were used to ensure system stability. The raw data of the samples and reference compounds including study protocols have been deposited in the open metabolomics database MetaboLights *via* the metadata annotation tool Isatab to enable efficient re-use of the datasets, such as in metabolomics cross-study comparisons or data fusion exercises.

## Background & Summary

Liquid chromatography coupled to tandem mass spectrometry (LC-MS/MS) enables capturing of metabolic snapshots at discrete time intervals^1^. Models can be built inspecting changes in metabolic trajectories over time for a group of closely related organisms distinguished by the characteristic properties under investigation^2^.

The ripening behaviour of *Solanum lycopersicum* and three ripening-inhibited mutants was explored using a LC-MS/MS assay (Table 1). The plant material was harvested in five to ten day intervals up to the point of ripening and daily after. Each genotype was grown in triplicate. Each block of 52 plant samples was measured using a group-randomization setup with interspersed quality controls: blank injections (solvent), pooled samples (mix, see table below), and aliquots of a pooled in-house standard tomato reference (see Methods). Randomization groups were defined by day of harvest.

The study resulted in a metabolic data set about the ripening behaviour of wild-type and mutant tomato, which completely abolish the normal ripening process (Data Citation 1)^3^. A total of 219 and 225 samples (including controls) were acquired in positive and negative ion mode respectively. Quality measures including positive and negative control samples as well as representative internal standards were taken into account during study design, facilitating data analysis and enabling filtering of unintended biological variation in the data. A total of 58 distinct reference standards relevant to plant metabolism were measured on the same instrumental setup to aid metabolite identification (Data Citation 2) and consequently model validation *via* biological interpretation.

Tomato is a model system to study ripening in fleshy fruits^4^. Many single gene mutants are well described, making it a suitable target to investigate various biological processes, including ripening, and integrate these^5^. Understanding metabolic changes underlying factors of ripening are essential to exploit regulatory mechanisms and improve fruit crop. Building on research of ethylene regulation in ripening, changes in metabolites in this climacteric fruit are of particular interest because of the link between ethylene biosynthesis and central metabolic pathways^6^.

By making this data set publically available, it lends itself to applications in the biochemistry of fruit ripening, metabolic cross-study comparisons or investigations into reproducibility of data analysis in metabolomics. The presence of standard reference files and complex biological data files—acquired on the same LC-MS/MS system under identical conditions—also make it highly useful for exercises around data analysis and interpretation. Other studies about the ripening behaviour of tomato include the multi-platform metabolomics analysis by Carrari *et al.* ^7^, Perez-Fons *et al.* ^8^ as well as others^9–12,9–12,9–12,9–12^ that could be used for combined analysis.

## Methods

### Plant material

Wild-type *Solanum lycopersicum* (Ailsa Craig, AC^++^) and three ripening inhibited AC^++^ mutants were used in this study: *ripening inhibitor* (*rin*), *non-ripening* (*nor*) and *Colourless non-ripening* (*Cnr*) mutations. The plants were grown in 24 cm-diameter pots in M3 compost (Levington Horticulture, Ipswich, and Suffolk, UK) and watered daily under standard greenhouse conditions. Developing fruit were sampled in five to ten day intervals (10, 15, 20, 30, 40 days post-anthesis) and daily from Breaker (49, 50, 51, 52, 53, 54, 55, 56 days post-anthesis). Breaker fruit were defined as those showing the first signs of ripening-associated colour change from green to orange. Non-ripe mutants were taken at day 49 as equivalents to breaker WT fruits. All plant samples were taken at the same time each day, frozen in liquid nitrogen, and stored at −70 °C until required.

### Sample preparation

Stock standard solutions were prepared for the analytical reference standards at a concentration of 1,000 μg/ml in 20/80 HPLC analytical grade Ethanol/Water and then diluted 10× for injection.

Tomato samples were subjected to an untargeted metabolite analysis by LC-MS/MS of polar extracts. Approximately 30 mg of dried tomato tissue was extracted with HPLC analytical grade Ethanol/Water 20:80. The polar extracts were diluted 10:1 with water and injected underivatised. Samples were acquired in three batches on 17, 20, and 21 September 2010 in positive ion mode and on 23, 24, and 27 September 2010 in negative ion mode.

### Chromatography

All samples were run on a Waters Acquity® UPLC system (Waters Corporation, USA), HSS T3 150×2 mm, 1.7 μm particles, UPLC column at 30 °C oven temperature. The solvents used for the assay consisted of 0.2% Formic Acid (Solvent A) and 98/2/0.2 Acetonitrile/Water/Formic Acid (Solvent B). Gradient [time (min)/%B] starting at flow rate 0.25 ml/min: 2.5/0, 7.5/10 (flow rate to 0.4 ml/min), 10.0/100, 12.0/100, 18.0/0, 25.0/0. Aliquots of 2 μl were injected.

### Mass spectrometry

The compounds were detected using a Thermo LTQ Velos Orbitrap mass spectrometer operating in positive and negative Electrospray ionization (ESI) mode at a resolution of 30,000 with a scan range from 85–900 *m/z* and 95–900 *m/z* respectively. MS/MS spectra were obtained in a data dependent manner through higher-energy collisional dissociation (HCD, normalized collision energy: 50.0) at a resolution of 7,500: The two most intense mass spectral peaks detected in each scan were fragmented to give MS2 spectra (100–900 *m/z*). Full scan data was acquired in FT (accurate mass) mode, MS/MS spectra were acquired in centroid mode. The LTQ Velos Orbitrap used the Xcalibur control software version 2.1.0 for data acquisition. Reference standards were acquired using the same protocol and experimental setting.

### Reference and internal standards

Reference standards were commercially purchased from Fluka Analytical, Sigma-Aldrich, and C/D/N Isotopes or prepared in-house. Internal standards for reference tomato aliquots comprised the following (final concentration): Citric acid-d4 (1,000 μg/ml), L-Alanine-d4 (200 μg/ml), Glutamic acid-d5 (200 μg/ml), and L-Phenyl-alanine-d5 (100 μg/ml). Pooled in-house standard tomato reference was prepared from the shop bought Angelle variety: mashed up in bulk and aliquoted out following the protocol outlined above.

### Data processing and transformation

Non-targeted LC-MS/MS vendor raw data files were converted to the open source format mzML using the program ProteoWizard^13^. Vendor-based peak picking was enabled for MS^1^. The resulting mzML files were subsequently processed with MassCascade^14^ in KNIME^15^, the open source Konstanz Information Miner environment: features were extracted with ±5 p.p.m. mass accuracy, smoothed using a third order polynomial and deconvoluted using a modified Bieman algorithm^16^. Noise reduction was firstly carried out by removing ion signals that are not consistent across six adjacent scans^17^ and secondly via a Durbin-Watson criterion set to a threshold of 2.8^18^. The workflow including all settings is available at MyExperiment^19^ under this article’s title. Obiwarp^20^ was used for cross-sample alignment before signals below an intensity threshold of 10,000 were filtered out. The maximum percentage of missing values per group and feature was set to 10% to account for limits in detection while ensuring that features used for statistical analysis are at least consistently present in 90% of the samples. Lower and higher percentages of missing values were found to decrease and increase the number of features respectively, without significantly affecting subsequent data validation. Overall, differences between groups were less pronounced with an allowed missingness of 0% and above a missingness value of 20%, most likely due to an increase in the sparsity of the feature matrix. Gaps in the resulting sample by feature matrix were either backfilled or—if missing—imputed using a readily available PCA-based approach^21^. In contrast to naïve methods (e.g., mean replacement), PCA-based gap filling takes the natural variance of the data into account and has been shown to give good results^22^. Statistical analysis was carried out in the statistical programming environment R, version 3.0.2. Total signal intensity normalization and Pareto scaling were used for data pre-treatment.

## Data Records

All samples used in this study have been submitted to MetaboLights^23^ at the European Bioinformatics Institute (EMBL-EBI). Each MetaboLights entry contains protocols about sample collection, extraction, chromatography, mass spectrometry, metabolite identification, and data transformation. The study was metadata tagged using the Investigation/Study/Assay (ISA) suite^24^. ISA uses tab-separated text files to store the experimental information. In addition, identified metabolites were stored in mzTab^25^ compatible tab separated files provided by the MetaboLights’s Isacreator plug-in extension.

### Data record 1

Data Citation 1 contains the study samples: 442 LC-MS/MS files (.mzML, 64-bit) acquired in continuous mode: 219 in positive and 223 in negative ion mode.

Consistent file names are composed of: <acquisitionDate>_<runId>_<sample>_<sampleTime>.

### Data record 2

Data Citation 2 contains the reference standards: 71 LC-MS/MS files (.mzML, 64-bit) acquired in continuous mode: 43 in positive and 28 in negative ion mode. Chemical names are used as file names and linked to the ChEBI database^26^. Additionally, putative *in silico* generated fragment structures for MS^2^ are collated in individual mzTab files for 34 out of 43 samples acquired in positive ion mode to aid interpretation. No MS2 spectra are available for the remaining 9 samples.

## Technical Validation

**Figure 1:**
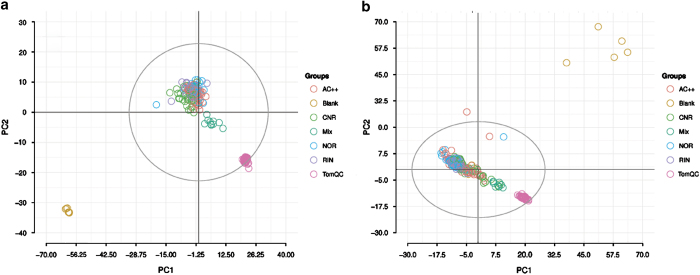
Principal Component Analysis of the data sets acquired in positive (**a**) and negative (**b**) ion mode. The first two principal components are shown. The data are coloured by group. Total signal normalization and Pareto scaling was applied to both data sets. Quality controls—blanks, mixed, and aliquots of reference standard tomato samples—cluster distinctively outside the main group. Blanks are well outside Hotelling’s T^2^ confidence region (95%, indicated as grey circle).

In mass spectrometry experiments, acquired data needs to be checked for unintended systematic and random factors that may distort analysis. These factors can result from the biological or experimental level and can be detected through good experimental design. Quality controls consisting of blanks, mixed samples, and standard tomato samples were used to assess and to validate the experimental design. The stability of the experimental system was inspected using principal component analysis (PCA) on the processed sample by feature matrices of the two data sets, acquired in positive and negative ion modes (Fig. 1). PCA is a well-established technique that can be used to provide a first overview of the data with regard to questions related to high variance, e.g., sample clusters, trends, and outliers. In all of the models the total signal is normalized and aligned signals Pareto scaled. As shown in Fig. 1, quality control samples cluster distinctively, indicating absence of significant non-biological induced variation in the study. *Nota bene*, PCA of the batches in the absence of quality controls shows that samples acquired on 17 September show strong batch variation compared to samples acquired on 20 and 21 September; this is very common and expected^27,28^.

Ion traces from internal standards were extracted from reference tomato aliquots after data processing and used to validate the consistency of the data set. Tables 2 and 3 summarize the results of the ‘positive’ and ‘negative’ data set. L-Alanine-d4 was not ionized in negative ion mode and consequently not detected in the negative mode.

The consistency of the retention times and abundances of the internal standards across samples—together with the clusters observed in the PCA—indicate little technical sample-to-sample variation^29^.

## Usage Notes

This data set can be used for data-driven cross-tool comparisons in metabolomics, investigating the effects of data processing, e.g., peak picking and deconvolution, and analysis of statistical models and subsequent biological interpretation. Open source tools and databases can be used to support and demonstrate efforts within the metabolomics community, driven by COSMOS^30^ as well as recent initiatives in the metabolomics society. Such a comparison can include feature extraction and cross-sample alignment by tools such as MzMine2^31^, MassCascade, and XCMS^32^ and subsequent exploration of interpretability and predictability of multivariate models build on different data processing approaches and tools. Additionally, effects of data pre-treatment methods such as scaling and normalization could be investigated with regard to metabolite ranking on biological importance according to developed models used.

With regard to ripening, the monogenic mutants (*nor, rin* and *Cnr*) can be compared to the wild-type AC^++^ to identify and investigate metabolites correlated to differences between the genotypes. This comparative analysis can involve Partial Least Squares Discriminant Analysis (PLS-DA) and S-plots to highlight statistically reliable features that are strongly correlated with the models between the individual mutants and wild-type. Knowledge of identified features, i.e. metabolites, is essential for metabolic modelling and exploration of regulatory mechanisms.

The data set can also contribute to the generation of a standard metabolome of tomatoes: submission of high quality data sets of key organisms such as *Solanum lycopersicum* into public databases, enable the community to gradually collect and subsequently discover the metabolome of a species. This study features metabolomics data from four different varieties of tomato that are semantically annotated, complete, and fully publicly available.

## Additional information

**How to cite this article:** Beisken, S. *et al.* Metabolic differences in ripening of *Solanum lycopersicum* ‘Ailsa Craig’ and three monogenic mutants. *Sci. Data* 1:140029 doi: 10.1038/sdata.2014.29 (2014).

## Supplementary Material



## Figures and Tables

**Table 1:** Summary of study samples.

**Name**	**Group**	**Description**
AC^++^	Wild type (WT)	Ailsa Craig variety
NOR	Monogenic mutant	Ailsa Craig near isogenic lines containing the Non-ripening mutation
RIN	Monogenic mutant	Ailsa Craig near isogenic line containing the ripening inhibitor mutation
CNR	Monogenic mutant	Ailsa Craig near isogenic line containing the Colourless non-ripe mutation
Mix	n/a	Pooled sample of AC^++^, NOR, RIN, CNR, and TomQC
TomQC	n/a	Standard in-house tomato aliquots
Blank	n/a	Blank sample with solvent
The table contains the sample name, its group, and description.		

**Table 2:** Summary of Internal standards in the ‘positive’ data set.

**Name**	**PubChem ID**	**[M+H]** ^**+**^	**Retention time**	**Average abundance**	**Standard deviation**
Citric acid-d4	16213286	197.0594	244 s	0.3172	0.061
L-Alanine-d4	12205373	94.0804	84 s	0.7830	0.087
Glutamic acid-d5	56845948	153.0919	91 s	0.4229	0.368
L-Phenyl-alanine-d5	13000995	171.1175	414 s	7.0474	0.543
The chemical name, PubChem compound identifier, main adduct mass-to-charge ratio, retention time, averaged abundance, and standard deviation is shown for the four deuterated standards.					

**Table 3:** Summary of Internal standards in the ‘negative’ data set.

**Name**	**PubChem ID**	**[M+H]** ^**+**^	**Retention time**	**Average abundance**	**Standard deviation**
Citric acid-d4	16213286	195.0449	260 s	0.0183	0.005
Glutamic acid-d5	56845948	151.0771	91 s	0.4402	0.028
L-Phenyl-alanine-d5	13000995	169.1033	417 s	0.0613	0.022
The chemical name, PubChem compound identifier, main adduct mass-to-charge ratio, retention time, averaged abundance, and standard deviation is shown for the three deuterated standards. L-Alanine-d4 was not detected in negative ion mode.					
